# 3D microtumors *in vitro* supported by perfused vascular networks

**DOI:** 10.1038/srep31589

**Published:** 2016-08-23

**Authors:** Agua Sobrino, Duc T. T. Phan, Rupsa Datta, Xiaolin Wang, Stephanie J. Hachey, Mónica Romero-López, Enrico Gratton, Abraham P. Lee, Steven C. George, Christopher C. W. Hughes

**Affiliations:** 1 Department of Molecular Biology & Biochemistry, UC Irvine, CA, USA; 2 Department of Biomedical Engineering, UC Irvine, CA, USA; 3 Laboratory for Fluorescence Dynamics, UC Irvine, CA, USA; 4 Department of Biomedical Engineering, Washington University in St Louis, MO, USA

## Abstract

There is a growing interest in developing microphysiological systems that can be used to model both normal and pathological human organs *in vitro*. This “organs-on-chips” approach aims to capture key structural and physiological characteristics of the target tissue. Here we describe *in vitro* vascularized microtumors (VMTs). This “tumor-on-a-chip” platform incorporates human tumor and stromal cells that grow in a 3D extracellular matrix and that depend for survival on nutrient delivery through living, perfused microvessels. Both colorectal and breast cancer cells grow vigorously in the platform and respond to standard-of-care therapies, showing reduced growth and/or regression. Vascular-targeting agents with different mechanisms of action can also be distinguished, and we find that drugs targeting only VEGFRs (Apatinib and Vandetanib) are not effective, whereas drugs that target VEGFRs, PDGFR and Tie2 (Linifanib and Cabozantinib) do regress the vasculature. Tumors in the VMT show strong metabolic heterogeneity when imaged using NADH Fluorescent Lifetime Imaging Microscopy and, compared to their surrounding stroma, many show a higher free/bound NADH ratio consistent with their known preference for aerobic glycolysis. The VMT platform provides a unique model for studying vascularized solid tumors *in vitro*.

Despite some recent successes cancer remains one of the leading causes of morbidity and mortality worldwide. New drugs come to market slowly, their development is enormously expensive, and many provide only modest improvements in quality or extension of life^1^. Part of the problem is that we lack good *in vitro* models, and so our understanding of tumor behavior in a complex 3D environment is limited and drug screens are often misleading.

Although tumors are complex three-dimensional (3D) structures with their own unique microenvironments, many studies ignore this complexity in the interest of simplicity and convenience. Studies are often performed using cell lines growing as two-dimensional (2D) monolayers in plastic dishes, and the role of matrix, stromal cells, and vasculature is ignored. 2D monocultures do not accurately mimic the complex interplay between tumor cells and their extracellular environment, and a growing body of evidence has emerged demonstrating differences in the phenotype of tumor cells when cultured in 3D^2^,^3^,^4^. For example, proliferation rates may differ, metabolic profiles may be altered, and dose-response curves for some drugs can differ by orders of magnitude^5^,^6^. This may result from targets not being expressed in 2D, from survival pathways being activated in 3D, or from contributions by stromal cells. Compounding these deficiencies is the fact that tumors receive nutrients and therapeutics through the vasculature, and this component of the tumor microenvironment has never been previously included in *in vitro* tumor models.

The growing use of tumor spheroids addresses some of these shortcomings – the cells are in a 3D environment and stromal cells may be present^7^. However, there is no vasculature and many tumor cell types, especially those with a highly invasive phenotype, do not readily form spheroids, and so cannot be assayed in these systems. Patient-derived xenograft (PDX) models maintain the tumor microenvironment^8^, but are time-consuming, expensive, and limited by the supply of tissue.

To address the concerns with current tumor models we have developed a microphysiological system that incorporates human cells in a 3D extracellular matrix (ECM), supported by perfused human microvessels^9^,^10^,^11^,^12^. The vessels are enveloped by perivascular cells, have physiologic flow, and deliver nutrients to the tissue. We refer to this “base” tissue as a vascularized micro-organ (VMO). The microfabricated platform is transparent, allowing for non-invasive optical imaging of the tissues. We have now incorporated tumor cells into the VMO platform to create vascularized microtumors (VMTs). Growth of the VMTs can be tracked through fluorescent protein expression, and fluorescence lifetime imaging microscopy (FLIM) can monitor their metabolic status^13^. We find that both the tumors and the vasculature are sensitive to standard-of-care chemotherapeutics, that the tumors show considerable metabolic heterogeneity, that their metabolic state differs significantly from the stroma, and that this can be altered by chemotherapeutic drugs. The VMT platform is thus an ideal model for studying tumor-vasculature interactions, tumor metabolism, and response of tumor cells and vasculature to anti-cancer drugs.

## Results

### Establishment of Vascularized Micro-Organs

**Figure 1:**
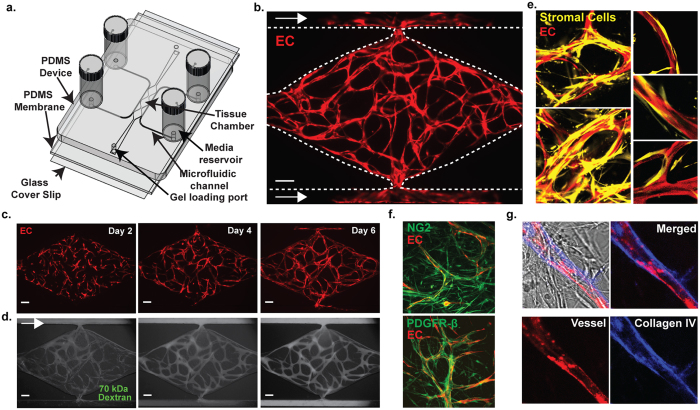
Establishment of Vascularized Micro-Organs (VMOs). (**a**) A schematic of the microfluidic platform. The VMO platform consists of a thick layer of PDMS containing patterned tissue chambers and microfluidic channels, bonded to a thin PDMS membrane and a glass cover slip. Three tissue chambers at the center are connected to two adjacent channels by two capillary burst valves that retain the mixture of cells and ECM inside the chambers. At the two ends of the tissue chambers are two gel loading ports, through which is introduced the cell-ECM suspension. Four media reservoirs are attached to the inlets and outlets of the microfluidic channels. (**b**) Representative tissue chamber with a fully-developed vascular network on day 7. Lentivirally-transduced EC (ECFC-EC, red) were visualized by confocal microscopy. Supporting stromal cells are unlabeled. EC migrate outward and anastomose with the microfluidic channels. (Scale bar 100 μm) (**c**) Representative time course of vascular network development (day 2, 4 and 6) (scale bar 100 μm). (**d**) Representative time course of 70 kDa FITC-dextran perfusion through the vascular network on day 7. Inflow was top left and outflow bottom right. The vascular network is fully perfused within 15 minutes. EC were labeled with mCherry. (Scale bar 100 μm). (**e**) Confocal imaging of lentivirally-transduced EC (red) and stromal cells (yellow) reveals that many take up a perivascular position. High magnification views on the right. (**f**) Immunostaining for PDGFR-β and NG2 (both green). EC are expressing mCherry. (**g**) Collagen IV staining (blue) identifies basement membrane deposition.

The VMO platform allows for the reproducible creation of perfused vascular networks *in vitro*. These networks support physiologic flow and can deliver nutrients to surrounding tissues sufficient to support robust growth. The platform is constructed using standard photolithography from polydimethyl siloxane (PDMS)^12^ and consists of two outer microfluidic channels that act as arteriole (high pressure) and venule (low pressure), connected by three tissue chambers, into which is injected a slurry of ECM and cells (Fig. 1a). Over the course of 5–7 days EC self assemble into an interconnected network that anastomoses with the outer channels. EC migrate into the outer channels, forming a tight seal, and over time, line the surface of the channel. At this point flow through the device switches from interstitial to intraluminal. Flow is generated by a hydrostatic head (Fig. 1a). We routinely transduce EC with mCherry for easy visualization of the network (Fig. 1b). Vessel-like fragments appear within 2–3 days and a complete network is apparent by 5–7 days (Figs 1c and S1a). Perfusion of the vascular network with 70 kDa FITC-dextran reveals the vessels to be minimally leaky (Fig. 1d, Supplementary Video SV1). We calculate the degree of leakage by measuring the increase of fluorescence signal in the extracellular compartment over time and this gives a permeability of 4.5 × 10^−7^ cm/s for 70 kDa-dextran, and 1.2 × 10^−7 ^cm/s for 150 kDa-dextran (Fig. S1b), which are in line with values obtained for capillaries *in vivo*^14^.

Stromal cells are required for proper formation of the vascular networks^15^,^16^, and a subset of these consistently envelop the newly-formed vessels, forming tight appositions (Fig. 1e). These cells are both NG2^+^ and PDGFR-β^+^ (Fig. 1f), consistent with a pericyte phenotype^17^,^18^. Once established, the vessels rapidly lay down a collagen IV-rich basement membrane (Fig. 1g). The stromal cells also express multiple other extracellular matrix proteins, including Collagen I (Fig. S1c), that are required for lumen formation^16^,^19^.

### Establishment of Vascularized Micro-Tumors (VMTs) and response to drugs

**Figure 2:**
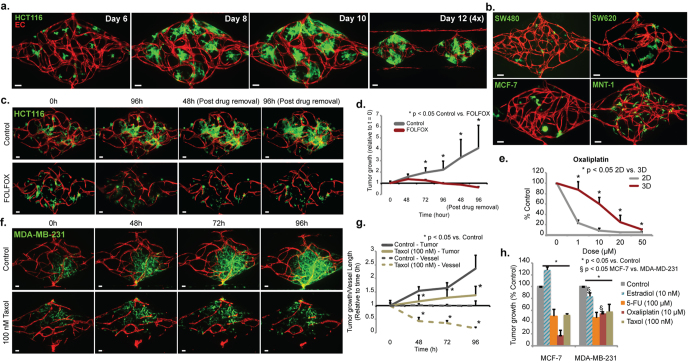
Establishment of Vascularized Micro-Tumors (VMTs). (**a**) Time course of HCT116 growth in the VMT. Lentivirally-transduced HCT116 (green) and EC (red) were visualized by confocal microscopy. Stromal/perivascular cells were unlabeled. (Scale bar 100 μm). (**b**) The VMT platform supports growth of multiple tumor cell types, including additional CRC (SW620, SW480), breast cancer (MCF-7) and melanoma (MNT-1). Images were taken between days 10 and 12. (**c,d**) Time course of HCT116 (green) response to FOLFOX (10 μM Folinic acid (leucovorin), 100 μM 5-FU, and 5 μM Oxaliplatin) in the VMT. Data are shown relative to t = 0, which is time of first exposure to drug (usually 6–8 days after initiation of culture). Cells were also monitored for 96 h after drug removal. Error bars show mean ± s.d of 3 replicates (n = 2) (p < 0.05 control vs treated FOLFOX). (Scale bar 50 μm). (**e**) Dose-response of Oxaliplatin on HCT116 cells. VMTs or cells growing in 2D were exposed to drug for 48 h, and tumor cell number and viability were assessed by fluorescence intensity measurement or XTT assay. Data are normalized to time zero of drug exposure and shown as percentage of control. Error bars show mean ± s.d (n = 3) (p < 0.05, 2D vs VMT). (**f,g**) Time course of MDA-MB-231 cells exposed to Taxol (100 nM). Data are normalized to first day of drug exposure. Error bars show mean ± s.d of 3 replicates (p < 0.05 control vs treated Taxol). (**h**) Differential effects of anti-tumor drugs on the breast cancer cell lines MCF-7 (ERα+) and MDA-MB-231 (triple-negative). VMTs were first exposed to E2/drugs between days 6 and 8 and cultured for an additional 96 h. Drugs were removed from the media at 48 h. Data are normalized to first day of drug exposure and are shown as percentage of control. Error bars show mean ± s.d (n = 2–4) (*p < 0.05 vs control; ^§^p < 0.05 MCF-7 vs MDA-MB-231).

We next incorporated tumor cells into the platform to create VMTs. The human colorectal cancer (CRC) cell line HCT116 was transduced to express GFP and introduced to the tissue chambers along with the EC, matrix and stromal cells. By day 6, when the microvasculature had formed, the tumor cells had proliferated to form small spheroids (Fig. 2a). Perivascular cells wrapped around a subset of the microvessels (Fig. S2) and vessels were found within and around the tumor spheroids (SV2). Tumor spheroids were often found in close apposition to microvessels (Fig. S2), consistent with tumor behavior *in vivo*^20^. Perfusion of the tumor-associated vascular network was confirmed using fluorescently-tagged dextran (Fig. S3). In addition to HCT116 we tested two other CRC lines (SW620 and SW480), two breast cancer lines (MCF-7 and MDA-MB-231), and a melanoma cell line (MNT-1) (Fig. 2b,e). In each case a vascular network formed that supported growth of the tumors. Interestingly, the tumors showed reproducibly different patterns of growth – SW480 and MCF-7 grew as tight colonies, whereas MDA-MB-231 and MNT-1 showed considerably more diffuse, invasive growth patterns (Fig. 2b). We also saw significant differences in growth rate, vascular development and collagen synthesis in each VMT model (Fig. S4), suggesting that each tumor cell line may be uniquely remodeling its microenvironment.

To test whether CRC VMTs respond to standard anticancer therapies we screened several FDA-approved drugs and drug combinations, including FOLFOX (5-FU, Leucovorin and Oxaliplatin), the standard-of-care chemotherapy for CRC. By 48 h FOLFOX had significantly reduced tumor growth compared to control (Fig. 2c,d), and by 96 h the tumors had regressed to below their starting volume. Importantly, even after drug removal the suppressive effect continued, confirming the cytotoxic rather than cytostatic effect of FOLFOX treatment. Also of note is the observation that the vascular network is largely unaffected, a finding we address in greater detail below.

We also tested as single agents several drugs with differing modes of action: 5-FU is a cytotoxic, cell-cycle–specific agent; Oxaliplatin is a cytotoxic platinum analog, and cell-cycle–nonspecific agent that induces DNA damage; Vincristine is a cell-cycle-specific M-phase agent; and, Sorafenib, is a small MW multikinase inhibitor that targets the vascular endothelial growth factor receptor (VEGFR) family, and platelet-derived growth factor receptor-β (PDGFR-β). It also inhibits the serine/threonine kinase Raf, which makes it useful in targeting tumors, such as HCT116, that carry the oncogenic KRAS mutation. To model a clinical regimen, drugs were added at concentrations that mirror patient plasma trough levels, and were removed after 48 h. VMTs were then cultured for an additional 2 days. All four drugs significantly reduced tumor growth, and when compared to data from 2D cultures, dose response curves showed a significant right shift (higher IC50 values), indicating drug resistance in the VMTs (Figs 2e and S5a,b.). It is unlikely that partition of drugs into PDMS is an issue in our device as there is a constant and relatively high convective flow through the microfluidic channels. The Peclet number represents the ratio of mass transport by convection to diffusion and in our device is equal to ~6.5 × 10^3 ^for a small molecular weight drug such as Sorafenib. Thus, although there may be some adsorption to the PDMS as the medium flows through the outer channels, this will not significantly reduce the concentration of the drug that reaches the tumor cells. In addition, protein adsorption and EC lining of the channels further limits loss of drug to the PDMS^21^,^22^.

There is considerable diversity in responses to drugs between tumors from different organs, and even within tumors from the same organ^20^. Responsiveness depends on mutational burden, gene expression patterns, and on the local microenvironment. In light of this we also tested two additional CRC lines as well as two breast cancer lines. The two CRC lines SW480 and SW620 are derived from the same patient, but from the primary site and from a lymph node metastasis, respectively. The lines have a largely overlapping mutation profile that differs from HCT116. We noted clear differences in response to a panel of drugs, with SW620 being significantly more drug resistant than HCT116 (Fig. S5c,d). Cells carrying p53 mutations are known to be relatively resistant to the apoptosis-inducing drug 5-FU, and we see this reproduced in the VMTs where SW620 (p53 mutant) are significantly more resistant to 5-FU than HCT116, which are wild-type for p53 (Fig. S5c,d).

We next examined the response of the triple-negative breast cancer line MDA-MB-231 to the standard-of-care chemotherapeutic Taxol – a mitotic disrupter. When left untreated for 96 h, the tumor cells grew rapidly and were highly invasive (Fig. 2f,g and compare to HCT116 in panel 2c). In contrast, cells treated with Taxol showed greatly reduced migration and growth. As expected, tumor vasculature was also disrupted in response to Taxol, in addition to its direct effects on tumor cells (Fig. 2f,g). MCF-7 cells (Estrogen Receptor (ER) alpha (α)+; Progesterone Receptor (PR)+) were similarly responsive to Taxol (Fig. 2h). We also examined the response of both cell lines to additional currently-used chemotherapeutic drugs as well as their response to physiological concentrations of 10 nM Estradiol (E2), which is important in breast cancer hormone-therapy. We found that whereas both cell lines are sensitive to Oxaliplatin, 5-FU and Taxol, they respond differently to E2 (Fig. 2h). As expected, MCF-7 cells, which are ERα+, showed an increase in tumor proliferation in response to E2, whereas the growth of MDA-MB-231 (ERα− and ERβ+) was moderately inhibited by the presence of the hormone (Fig. 2h). This result is in agreement with the proposed use of E2-agonists for the treatment of triple negative breast cancer^23^. A third tumor type – melanoma (MNT-1 (Fig. 2b) and A375 cells (not shown)) – also grows well in the VMT platform, reinforcing the utility of this system for the study of multiple tumor cell lines.

### Vasculature as a drug target

**Figure 3:**
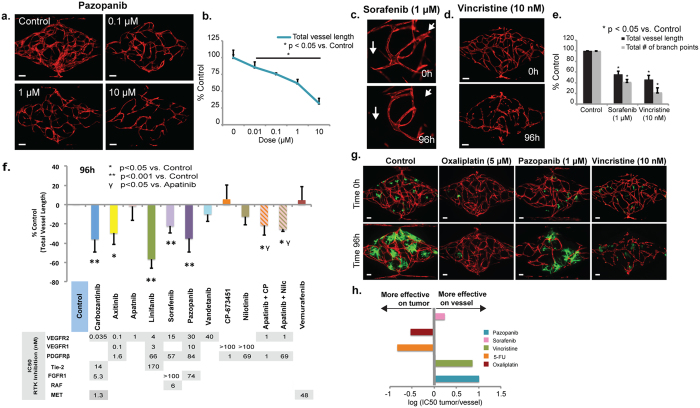
Vasculature as a drug target. (**a,b**) Effect of Pazopanib on vascular network integrity. VMOs were exposed to drug from day 4 to 6. Data are normalized to day of first drug exposure and are shown as percentage of control. Error bars show mean ± s.d (n = 3) (p < 0.05 vs control). (**c–e**) Effect of the anti-vascular agents, Sorafenib (1 μM) and Vincristine (10 nM) on total vessel length and total number of branch points in the VMO. VMOs were exposed to the drug between days 5 to 7 and cultured for an additional 96 h. Error bars show mean ± s.d (n = 3) (p < 0.05 vs control). Sorafenib induces vessel regression (**c**), whereas vincristine induces vascular damage (**d**). (**f**) Effect of 10 different RTK inhibitors on vascular network integrity. VMOs were exposed to drugs between days 5 and 7 and cultured for an additional 96 h. Data are normalized to first day of drug exposure and are shown as percentage of control. Error bars show mean ± s.d (n = 3- 5) (*p < 0.05 vs control; **p < 0.001; ^γ^p < 0.05 vs Apatinib alone). (**g**) Anti-cancer drugs that target tumor, vasculature, or both. HCT116 tumor cells in a VMT exposed to Pazopanib (1 μM), Oxaliplatin (5 μM) and Vincristine (10 nM). Images before and after drug exposure are of the same VMT (Scale bar 100 μm). (**h**) Summary of the relative IC50_tumor_/IC50_vessel_ values (from (**g**) and data not shown) plotted on a log scale. Drugs extending to the right are more effective, relatively, on vessels, whereas those on the left are more effective on tumor cells.

A key feature of tumor progression is the angiogenic switch^20^. The formation of new blood vessels in and around the tumor can be driven by a number of tumor-derived factors, a key family being the VEGFs. Targeting these growth factors and their receptors has proven moderately successful in the treatment of several human cancers, including cervical^24^, glioblastoma^25^, and metastatic CRC^26^, especially in combination with more established anti-cancer agents. We therefore examined the effect of several anti-angiogenic drugs on stability of the vascular network. Importantly, we use Endothelial Colony-Forming Cell (ECFC)-derived EC in the VMT as these have been suggested to be a good model for tumor EC^27^. Pazopanib is one of a new generation of tyrosine kinase inhibitors, with potent activity against VEGFRs, and to a lesser extent, PDGFR. Addition of Pazopanib resulted in a dose-dependent decrease in total vessel length, total number of junctions and lumen diameter after 72 h of drug exposure (Fig. 3a,b). The multikinase inhibitor Sorafenib is a widely-used antiangiogenic agent, whereas the mitosis inhibitor Vincristine is known to behave as a vascular disrupting agent in the tumor microenvironment^28^,^29^. Exposure of vessels to these reagents clearly distinguishes their modes of action: in both cases we see a significant decrease in total vessel length and total number of branch points after 96 h of drug exposure (Fig. 3c–e). However, regression of newly-forming (angiogenic) vessels is observed only with Sorafenib, while complete disruption of the network is observed after treatment with Vincristine (Fig. 3c,d). We next compared 10 clinically-approved receptor tyrosine kinase (RTK) inhibitors known to target VEGFR2, PDGFR, Tie2 and/or FGFR1 among others, using a standard dose of 100 nM (Fig. 3f). Compounds such as Apatinib and Vandetanib that target only VEGFR2 were not effective in disrupting the vascular networks, whereas compounds that inhibit both VEGFR2 and PDGFR, such as Axitinib and Pazopanib, were more effective. In line with this, combined use of PDGFR inhibitors and Apatinib increased the effectiveness of the VEGFR2 inhibitor. Finally, we identify Linifanib and Cabozantinib as being most effective in this assay, quite possibly due to their activity against Tie2, in addition to their inhibition of VEGFR2 and PDGFR. We also found that the effect of Linifanib persisted longer after removal than for other drugs tested (Fig. S6), in line with its success in intermittent dosing regimes in preclinical studies^30^.

Many drugs, including both Sorafenib and Vincristine, are thought to have dual effects in the tumor microenvironment, targeting both the tumor and the blood vessels that support it (see above). We looked for these dual effects in the VMT platform. We found that FOLFOX treatment reduces tumor growth by 70% while inhibiting vascular growth at most by 30% (Fig. S7). In contrast to FOLFOX, the antiangiogenic drug Pazopanib was more effective targeting vessels, while Oxaliplatin and 5-FU, not surprisingly, were more effective against tumor (Figs S8 and 3g,h). These results translate to IC50 ratios (tumor growth: total vessel length) of 10.7, 7.1, 0.3, and 0.15 for Pazopanib, Vincristine, Oxaliplatin, and 5-FU, respectively (Fig. 3h). For Sorafenib, which targets angiogenesis through the VEGFRs and PDGFR-β, and tumor growth and proliferation directly through Raf kinase, the ratio was closer to 1 (Figs S8 and 3h). The VMT platform can, therefore, distinguish between the multiple modes of action of currently-used RTK inhibitors.

### Tumor metabolism and metabolic response to drugs

**Figure 4:**
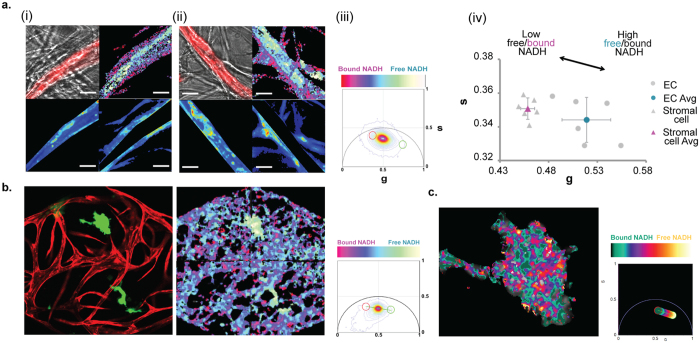
Fluorescence Lifetime Imaging Microscopy (FLIM) of VMTs. (**a**) Two different tissue chambers are shown in (i) and (ii). For each: EC (red) overlaid on the bright-field image (top left); 2PE-FLIM map of EC forming vessels and wrapped with perivascular cells (top right); Vessel and perivascular cell regions of interest (ROIs) were selected by manual masking and the average NADH phasor in each of these ROIs was calculated for ECs forming vessels (bottom left) and for the perivascular cells (bottom right), as described in Materials and Methods. Scale bar 20 μm. (iii) NADH FLIM phasor distribution used to create the free/bound NADH color scale. (iv) the average phasors for the ECs and the perivascular cells. Error bars show mean ± s.d of 6 replicates (n = 3, p < 0.05). (**b**) Left: confocal image of MCF-7 breast tumor cells (red) and vasculature (green). Right: 2PE-NADH FLIM map of the same VMT, and the NADH FLIM phasor distribution used to create the free/bound NADH color scale. (**c**) High resolution 2PE-NADH FLIM map of the tumor shown in (**b**) demonstrating metabolic heterogeneity, and the NADH FLIM phasor distribution used to create the free/bound NADH color scale.

Cellular metabolism is a potentially powerful biomarker for cellular state and response to drugs^31^,^32^. NADH is the major electron acceptor in glycolysis and electron donor in oxidative phosphorylation, and the rate of glycolysis in cells has been found to correlate strongly with the ratio of free to bound NADH^33^. This ratio can be determined by examining the rate of fluorescence decay of NADH, which is extended when NADH is protein-bound^34^. Fluorescence Lifetime Imaging Microscopy (FLIM) is a label-free imaging modality that when coupled with the phasor approach to data analysis can report on free/bound NADH ratios at single pixel resolution^13^,^33^. We first used FLIM to examine the metabolic profile of the vasculature in VMOs (Fig. S9a), and confirmed that potassium cyanide (KCN), a known blocker of the respiratory mitochondrial transport chain, induced a shift from bound to free NADH (Fig. S9b,c), in line with inhibition of OxPhos^35^. Interestingly, when we looked at high resolution (Fig. 4a) we found a higher relative level of free NADH in the EC (blue/white – see phasor plot in 4a(iii) for mapping) compared to either the perivascular cells investing the vessels or the interstitial stromal cells. This result suggests that EC are more glycolytic than stromal cells, which is consistent with published reports^36^,^37^. We also examined the metabolic profile of the vasculature in the presence or absence of flow, which limits nutrient delivery but not oxygen delivery (which diffuses freely through the PDMS). Under no-flow conditions the EC showed a decrease in free NADH (Fig. S10), suggestive of a shift away from a higher glycolytic rate (high glucose consumption/low ATP yield) to perhaps an increase in oxidative phosphorylation (lower glucose consumption/higher ATP yield), although the latter has not been confirmed.

As described above, cellular metabolism is a potentially powerful tumor biomarker and target. Cancer cells often rely on glycolysis, uncoupled from OxPhos and independent of local O_2_ concentrations, for energy production (known as aerobic glycolysis or the Warburg effect)^38^. We next asked whether the FLIM-phasor approach could be used in VMTs to interrogate tumor cell metabolism and the response of cells to chemotherapeutic challenge. As shown in Fig. 4b we found much higher NADH free/bound ratios in MCF-7 breast cancer cells (blue/white) relative to other cells in the surrounding microenvironment (red), including vascular and stromal cells. This suggests that the tumor cells are more glycolytic than the surrounding cells, which is consistent with their known preference for aerobic glycolysis. There is a growing interest in tumor heterogeneity, and in particular in metabolic heterogeneity within tumors^33^,^39^. High resolution FLIM imaging of just the tumor in (b) shows a clear pattern of high free NADH interspersed with areas of high bound NADH (Fig. 4c), consistent with distinct domains of metabolic activity, and in line with what has been seem in colon tumors *in vivo*^33^. Additional FLIM images of MCF-7 cells are shown in Fig. S11, where again, metabolic heterogeneity is clearly evident.

**Figure 5:**
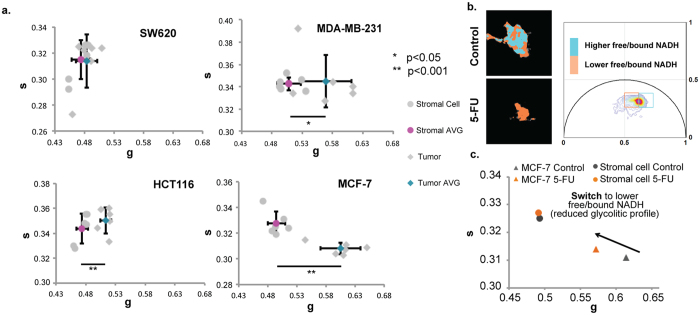
Heterogeneity of FLIM signatures between different tumors. (**a**) Phasor scatter plots comparing SW620, HCT116, MCF-7 and MDA-MB-231 to their surrounding stroma. Error bars show mean ± s.d (p < 0.01 for MCF-7 and HCT116; p < 0.05 for MDA-MB-231). (**b**) Top left: NADH FLIM map of MCF-7 cells treated with control or 5-FU (100 μM). Two colors, cyan and orange are used to depict two lifetime phasor distributions with cyan representing a higher free/bound NADH ratio, while orange shows a comparatively lower free/bound NADH ratio. Top right: phasor plot showing NADH phasor distribution. (**c**) Scatter plot showing the NADH phasors for the treated and non-treated conditions for the tumor and stroma shown in (**b**).

We next mapped the FLIM signatures of three additional tumor cell lines and compared these to the FLIM signature of their surrounding microenvironments (Fig. 5a). Similar to MCF-7, both MDA-MB-231 and HCT116 had a significantly higher NADH free/bound ratio than their surrounding stroma, whereas SW620 were indistinguishable. We next treated MCF-7 cells with 5-FU and compared the NADH free/bound ratio to that in untreated cells (Fig. 5b). Notably, we saw a strong decrease in the ratio of free/bound NADH in the treated cells, consistent with a slowed glycolytic rate. It should be noted that 5-FU induces apoptosis. which also leads to high levels of bound NADH (red)^40^. Importantly, the metabolic profile of the stroma remained unchanged in response to 5-FU (Fig. 5b). Taken together, these data suggest a hierarchy of free/bound NADH (and potentially aerobic glycolytic rate) in the cells of the tumor microenvironment, from tumor (highest glycolytic rate/lowest OxPhos profile) to EC (moderate glycolytic rate) to stroma (lowest glycolytic rate/highest OxPhos profile), and furthermore suggest that inhibiting glycolysis may be an effective strategy to target tumor cells as well as their associated vasculature.

## Discussion

We have developed a vascularized micro-organ (VMO) platform that can be configured for tumor studies. The vascularized microtumors (VMTs) capture some of the complexity of *in vivo* tumors, including 3D structure, extracellular matrix, the presence of stromal cells and the delivery of nutrients and drugs through a perfused vascular network. Importantly, the microvessels are not pre-patterned as they are in several recently published models^41^,^42^, which allows for remodeling in response to physiologic cues or pharmacologic therapy. Despite the relative complexity of the tissue, the platform is simple, requiring no external pumps, tubing, or robotics. Fluid flow is driven by gravity and the device can be readily configured to a 96-well plate format. The vessels are invested with cells expressing pericyte markers and their permeability characteristics match vessels *in vivo*.

The platform supports multiple tumor types, including invasive cells, such as MDA-MB-231, that do not readily form spheroids. We observed significant differences in IC50 doses for multiple drugs when 2D cultures were compared to the 3D VMT platform, which compares well with previous work using 3D cultures^2^,^43^,^44^,^45^. We also suspect that the relative complexity of the microenvironment may contribute to these differences. It is important to note however, that the VMTs still lack some components of *in vivo* tumors, most notably, an immune component. While addition of macrophages to the VMT is trivial, incorporating an adaptive immune component will require sourcing of entirely autologous cells. These could be derived from the tumor, or generated from iPS cells. EC have previously been derived from iPS cells^46^,^47^ and so this goal may be within reach. Importantly, tumor cells with defined mutations retain their characteristic responses to chemotherapeutics in the VMT platform – SW620 cells, which carry a p53 mutation were considerably more resistant to 5-FU than HCT116, which express wild-type p53^48^. We have generated VMTs with six cancer cell lines so far, including three CRC lines, two breast cancer lines and a melanoma line, suggesting that the platform should be amenable to study of most cancer cell lines.

The development of new anticancer treatments includes identification of compounds that target cell signaling pathways involved in pathological EC proliferation, migration and morphogenesis^49^,^50^. We have shown that the microvessels in the VMT platform are sensitive to the effects of both anti-angiogenic (Pazopanib, Linifanib, Cabozantinib, Axitinib, etc.) and vascular disrupting agents (Vincristine, Taxol), thus the platform can be used to identify reagents that target tumor cells directly, or indirectly through effects on the vasculature. Interestingly, the hierarchy of drug effectiveness we see (Fig. 3F) matches well with data from zebrafish studies^51^. We have also noted enhanced angiogenic sprouting in the presence of tumor cells, as well as enhanced vascular leakage (unpublished observations).

A particularly exciting potential use for the VMT platform is in the study of metabolic changes in tumor and stroma in response to drug treatments. EC have previously been reported to show a preference for glycolytic activity^36^ allowing oxygen to diffuse into deeper proliferating tissues, and the higher relative level of free NADH compared to the surrounding stroma in the VMT platform supports this finding. We find that multiple tumor cell types (e.g. MCF-7, MDA-MB-231, HCT116) are shifted even more in the direction of free NADH (aerobic glycolysis) relative to the stroma, suggesting that there is a hierarchy of glycolytic rate from stroma (lowest), through EC, up to tumor (highest). There is a growing awareness of the potential for reducing tumor drug resistance through metabolic targeting^52^,^53^,^54^ and our data support the idea that reducing glycolysis may have some specificity for glycolysis-dependent tumors, and perhaps, their associated vasculature. In support of this we found that 5-FU, while severely disrupting the NADH free/bound ratio in MCF-7 tumor cells had no effect on the stromal NADH free/bound ratio. Importantly, we also see strong metabolic heterogeneity in the tumors, consistent with previously reported *in vivo* studies^33^. There is a growing awareness that tumors may become drug-resistant, at least in part, through a metabolic switch to OxPhos (a reverse-Warburg effect), making the targeting of this a promising therapeutic approach. The VMT platform could prove ideal for identifying suitable drugs.

Future improvements to the VMT platform will include the incorporation of tumor-derived matrix and the use of plasma-containing medium that more fully reflects the composition of the fluid bathing tumor cells *in vivo*. Finally, the VMO platform is clearly suitable as a base for other organ systems, including for example, heart, liver, pancreas and the blood-brain barrier.

## Materials and Methods

### Design of the microfluidic platform

The device consists of 2 straight microfluidic channels separated by 3 diamond-shaped tissue chambers (1 × 2 × 0.1–0.12  mm). The channels are connected to 2 media inlets and outlets on each side. On top of each inlet and outlet, a large medium reservoir is attached to establish hydrostatic pressure (10 mm H_2_O) across the microfluidic channel and a 5-mm H_2_O interstitial pressure across the tissue chambers. The channels are connected on either side of the tissue chamber via a communication pore 50 μm wide. The pore is designed with a unique curved opening that mimics the capillary burst valve to prevent the fibrin gel from bursting into the microfluidic channels when seeding the device, while also bringing the gel boundary close to the straight microfluidic channels to facilitate anastomosis of the vascular network inside the issue chambers.

### Microfabrication of the PDMS microfluidic device

The microfluidic device is created using standard PDMS soft lithography. To develop the mold, a 100 μm layer of SU-8 is spin-coated onto Si-wafer (RCA-1 cleaned and 2% HF treated), following by a single mask photolithography step to pattern the tissue chamber, communication pores, and fluidic channels. The microfabricated SU-8 mold is silanized with trichlorosilane (C_8_H_4_Cl_3_F_13_Si) in a vacuum chamber. To fabricate the microfluidic device, an 8 mm-thick layer of PDMS (Dow Corning) is molded onto the SU-8 mold, degassed in a vacuum chamber, and allowed to solidify at 70 °C overnight. The PDMS microfluidic device is de-molded, hole-punched, and bonded to a 1 mm-thick membrane of PDMS attached to a microscope coverslip slide (Corning). Medium reservoirs are attached to the bonded device using PDMS and allowed to solidify at 70 °C overnight. The device is subsequently sterilized by autoclaving at 120 °C before use.

### Cell culture

Human endothelial colony forming cell-derived ECs (ECFC-ECs) are isolated from cord blood and expanded on gelatin-coated flasks in EGM-2 (Lonza). The methods were carried out in accordance with the approved guidelines and with approval from UC Irvine’s Institutional Review Board. ECFC-ECs are transduced with lentivirus to express fluorescent proteins (mCherry, or GFP) and used between passages 4–7. Normal human lung fibroblasts (NHLFs) are purchased from Lonza, cultured in DMEM (Corning) containing 10% Fetal Bovine Serum (FBS, Gemini Bio Products). While the cells in different lots all express NG2 and CD90 there are considerable differences between lots. We select lots for further studies based on higher NG2 and CD90 expression as these perform better as perivascular cells. We use them between passages 5–8. Colorectal cancer cell lines (SW620, SW480, and HCT116), breast cancer cell lines (MDA-MB-231, and MCF-7), and a melanoma cell line (MNT-1) were obtained from UC Irvine’s Chao Family Comprehensive Cancer Center and transduced with lentivirus to express fluorescent proteins (mCherry, GFP, or Azurite Blue). All cancer cell lines are cultured in 10% FBS DMEM. All cell types are grown in a 37 °C, 5% CO_2_ and 20% O_2_ incubator in 100% humidified air. Lentiviral constructs to express mCherry and GFP (LeGO-C2, LeGO-V2) were gifts from Boris Fehse (Addgene plasmids # 27339 and 27340), and lentiviral construct to express Azurite Blue was a gift from Pantelis Tsoulfas (Addgene plasmid # 36086).

### Seeding the devices

Fibrinogen solution is prepared by dissolving 70% clottable bovine fibrinogen (Sigma-Aldrich) in 1× Dulbecco’s Phosphate Buffered Saline with Ca^2+^/Mg^2+^ (LifeTechnologies) to a final concentration of 10 mg/mL. NHLFs and ECFC-ECs are harvested and resuspended in fibrinogen solution, each at a concentration of 5 × 10^6 ^cells/mL. To create a tumor microtissue, cancer cells are then harvested and resuspended in the mixture at a concentration of 200,000 cells/mL. The final cell-matrix suspension is mixed with thrombin (50 U/mL, Sigma-Aldrich) for a final concentration of 3 U/mL, quickly seeded into the microtissue chambers, and allowed to polymerize in a 37 °C incubator for 15 minutes. Laminin (1 mg/mL, LifeTechnologies) is then loaded into the microfluidic channels through medium inlets and incubated at 37 °C for an additional 15 minutes to stimulate ECFC-EC anastomosis with the microfluidic channels. After incubation, culture medium (EGM-2, Lonza) is introduced into the microfluidic channels and subsequently medium reservoirs to establish a 5-mm H_2_O interstitial pressure across the tissue chambers. Medium in reservoirs is changed and leveled every other day to maintain interstitial flow. Anastomosis of the vascular network is confirmed by introducing 70 kDa FITC or Rhodamine B-dextran (LifeTechnologies) to the medium reservoir on day 7 post-embedding, and visualization using fluorescent microscopy.

### Drug exposure in the device

To assess drug screening experiments, cells in the microfluidic device were exposed to reported *in vivo* plasma concentrations of different FDA approved anti-cancer drugs or a standard dose of 100 nM. 5-Fluoruracil (5-FU), Vincristine, Sorafenib and Taxol were obtained from the NIH-MPS Training Compound Collection at Evotec. Oxaliplatin, Pazopanib, Linifanib, Apatinib and CP-673451 were purchased from Selleck Chemicals. Vemurafenib, Vandetanib, Cabozantinib and Sorafenib were obtained from National Cancer Institute (NCI) plate sets. All compounds were dissolved in dimethyl sulfoxide (DMSO) and added in the medium with less than 0.01% DMSO. For hormone response in breast cancer cell lines, Estradiol (Sigma-Aldrich) was used at physiological plasma concentration. Estradiol was dissolved in 100% ethanol and added in the medium with less than 0.001% Ethanol. For a typical screening assay, after 5–8 days of cells cultured in the microdevice, media from the device is replaced by media containing the drug at the desired concentration, and delivered through the microfluidic channels using the hydrostatic pressure gradient.

### Time-lapse image sequences and time course image analysis

For tumor and vessel quantification within the microfluidic device, and time-lapse image sequences, time course images were taken using a SPOT Pursuit High Speed Cooled CCD camera (SPOT Imaging). For tumor quantitative analysis, fluorescent intensity of mCherry, GFP, or Azurite Blue was analyzed using ImageJ software (National Institute of Health). Vessel fluorescent intensity of mCherry was used for vessel quantitative analysis. Total vessel length and total number of branch points was performed using AngioTool (National Cancer Institute). Vessel diameter were determined using the MATLAB subroutine, RAVE, developed by Seaman *et al*.^55^. Final values were normalized to time zero of drug exposure. Three replicates (microtissue chambers) were examined per experiment. Confocal imaging of fluorescence reporters was performed on a Zeiss LSM 710 microscope (Carl Zeiss, Jena, Germany) using a 20x air objective, 0.5 N.A. (EC Plan-Neofluor, Carl Zeiss, Oberkochen, Germany). GFP was excited with 488 nm and signal collected between 493–581 nm. mCherry was excited with 561 nm and emission collected between 578–696 nm. mRFP was excited with 561 and emission collected between 582–754 nm. Azurite Blue was excited at 405 nm and emission collected between 410–585 nm. Image acquisition and post processing was done using Zen software (Zeiss, Germany). Where adjustments were made to images these were performed on the entire image, and all images in that experimental group were adjusted to the same settings. To reduce confusion, some images have been re-colored to maintain consistent color-coding throughout the paper. Thus vessels are shown as red and tumor cells as green, although in some cases EC were expressing GFP and the tumor cells were expressing mCherry (or mRFP). We have seen no differences in cell behavior when they express GFP vs mCherry or mRFP.

### Vascular permeability quantification

For vascular permeability quantification, medium is replaced by DPBS with similar hydrostatic pressure profile, and the device is positioned onto a microscope stage. 70 kDa-FITC or 150 kDa FITC-dextran (Sigma-Aldrich) is introduced to the reservoir with highest hydrostatic pressure to a final concentration of 50 μg/mL, and allowed to perfuse through the microfluidic channel. An image of background fluorescent intensity is acquired prior to addition of the dye. After allowing FITC-dextran to fully perfuse the microvascular network (15 minutes), time-lapse images are acquired every 15 minutes for a time course of 90 minutes. The diffusive component of the solute permeability coefficient *P* is calculated using the equation previously described^56^ by quantifying the background average fluorescence intensity (*I*_b_), the initial average fluorescence intensity (*I*_i_) step change after FITC-dextran influx reached equilibrium at the initial time point, and the final average fluorescence intensity (*I*_f_) of 12 central regions of tissue chamber. Three independent experiments for 70 kDa and 150 kDa permeability coefficient quantification are obtained for statistical analysis.

### Collagen I image acquisition

Second harmonic generation (SHG) signal was acquired using the microscopic system developed by the Laboratory of Fluorescent Dynamics (LFD) at UCI^57^. A Ti:Sapphire laser (Mai Tai, Spectra Physics, Irvine, CA) was used for two-photon fluorescence excitation, with a wavelength of 740 nm and an incident power in the sample of 20 mW. The signal was collected using a long working distance water objective (LUMPlanFl 40x/0.80 W, Olympus, Tokyo, Japan). The SHG signal was obtained using a 320 nm- 390 nm bandpass filter. Images were taken every 5 μm through the sample with a depth of view of 740 μm. Data acquisition was performed in SimFCS (software developed by the LFD). The images were analyzed with the free ctFIRE MATLAB version (http://loci.wisc.edu/software/ctfire), extracting the information corresponding to fiber length of 3 replicates for every time point.

### Immunofluorescent staining

For immunofluorescent staining, devices were fixed by flowing 4% paraformaldehyde (Sigma-Aldrich) for 2 h at room temperature, followed by an overnight PBS wash at 4 °C. Blocking, washing, antibody incubation, and nuclei staining steps are also conducted by flowing reagents through the microfluidic channels overnight at 4 °C. Collagen IV was observed by staining using rabbit anti-human Collagen IV polyclonal antibody (AbD-Serotec 2150-0150, Bio-Rad) followed by Alexa 408 Donkey anti-rabbit secondary antibody. Confocal imaging of stained collagen IV was performed on Zeiss LSM 710 microscope (Carl Zeiss, Jena, Germany) using a 20x air objective, 0.5 N.A. (EC Plan-Neofluar, Carl Zeiss, Oberkochen, Germany), excited with 405 nm and emission signal was collected between 410–585 nm. The vascular network was observed by staining with PE/Cy7 anti-human CD31 antibody (BioLegend). Perivascular cells were visualized using antibodies to NG2 (Millipore EMD/MAB5384) and PDGFR-β (Cell Signaling/3169) at 1:200 dilution. Secondary antibody was Alexa Fluor 488 (ThermoFisher), used at 1:300.

### Fluorescence lifetime image acquisition and analysis

Fluorescence lifetime imaging microscopy (FLIM) of reduced nicotinamide adenosine dinucleotide (NADH) was performed on a Zeiss LSM 710 microscope (Carl Zeiss, Jena, Germany) using a 20x air objective, 0.5 N.A. (EC Plan-Neofluar, Carl Zeiss, Oberkochen, Germany) with two photon excitation of 740 nm (titanium:sapphire MaiTai laser from Spectra-Physics, Mountain View, CA). Image scan speed was 25.21 μs/pixel and image size is 256 × 256 pixels. For NADH FLIM of the whole chambers, a 2 by 2 tile scan was performed. 460/80 nm bandpass filter was employed as emission filter and a photomultiplier tube (H7422P-40, Hamamatsu, Japan) was used for detection. FLIM data was acquired using A320 FastFLIM FLIMbox (ISS, Champaign, IL). For acquisition and FLIM data analysis SimFCS software, developed at the Laboratory for Fluorescence Dynamics (LFD, UC Irvine), was used. For analysis of FLIM data, the phasor approach was used. In this method, lifetime information from every pixel of the image is transformed into a phasor on the phasor plot to create the FLIM phasor distribution, as described previously^58^. By NADH FLIM phasor analysis, we can map the free to protein bound NADH distribution in the images which can be correlated to the metabolic status of the biological sample^13^,^33^.

### Cell viability assay

2D screening experiments in tumor cells were assessed using the colorimetric XTT-cell viability assay according to the manufacturer’s protocol (Sigma). Briefly, HCT116 were seeded into 96-well plates in 100 μL at plating densities of 5,000. Tumor cell density was selected according to the doubling time of the cell line to determine tumor cell proliferation. After cells were seeded, the plates were incubated at 37 °C, 5% CO_2_, 95% air and 100% relative humidity for 24 h prior to addition of drugs. For optimal comparison between 2D and 3D, the drug screening schedule in 2D was selected to match the one used in the microfluidic device. XTT absorbance values at 450 nm (corrected at 690 nm) were measured using a microplate spectrophotometer. Three replicates for each exposure concentration were examined. Results were expressed as percent cell viability for each concentration with respect to DMSO controls.

### Statistical analysis

Data represent mean ± s.d unless otherwise stated. The differences between experimental groups of equal variance were analyzed using Student’s t test. Estimated means, standard deviation, and significance levels were calculated using GraphPad software. Number of replicates is indicated in the legends. The level of significance was set at p < 0.05.

## Additional Information

**How to cite this article**: Sobrino, A. *et al*. 3D microtumors *in vitro* supported by perfused vascular networks. *Sci. Rep.* **6**, 31589; doi: 10.1038/srep31589 (2016).

## Supplementary Material

Supplementary Information

Supplementary Video S1

Supplementary Video S2
