# Supplementary material for: 3D microtumors in vitro supported by perfused vascular networks
Source: Sci Rep. 2016 Aug 23;6:31589. doi: 10.1038/srep31589 (PMC4994029; doi:10.1038/srep31589)
Supplement: Supplementary Information [file srep31589-s1.pdf]

3D microtumors *in vitro* supported by perfused vascular networks

Agua Sobrino *[1], Duc T. T. Phan *[1], Rupsa Datta[2,3], Xiaolin Wang[2], Stephanie J. Hachey[1], Mónica Romero-López[1,2], Enrico Gratton[2,3], Abe P Lee[2], Steven C. George[4] and Christopher C.W. Hughes[1,2]

Affiliations

[1]Departments of Molecular Biology & Biochemistry and [2]Biomedical Engineering, [3]Laboratory for Fluorescence Dynamics, UC Irvine, and [4]Department of Biomedical Engineering, Washington University in St Louis, USA.

Correspondence to CCWH (cchughes@uci.edu)

Contributions

* These authors contributed equally to this work.

Supplementary material

SUPPLEMENTARY FIG. 1

(a). Total vessel length over time, quantified using AngioTool. Error bars represent mean ± s.d of 3 replicates from a representative experiment ($p < 0.05$) ($n = 3$).

(b) Permeability coefficients of the vascular networks perfused with 70 kDa and 150 kDa FITC-dextran for 90 minutes. Error bars mean ± s.d ($n = 3$) ($p < 0.05$).

(c) Time course of collagen I increase in the VMO. Devices were fixed in 4% PFA at different time points and quantification was done using second harmonic generation imaging as described in Material and Methods. Error bars mean ± s.d of 3 replicates ($p < 0.05$).

SUPPLEMENTARY FIG. 2

Interactions between microvessels, stromal cells and HCT116 tumor cells. Many of the stromal cells take up a perivascular position. Lentivirally-transduced HCT116 (green), stromal/perivascular cells (yellow) and EC (red) were visualized by confocal microscopy (Scale bar 100 μm).

SUPPLEMENTARY FIG. 3

70 kDa fluorescently-labeled dextran perfusion through 3D VMT. Upper panels show a time course of 70 kDa FITC-dextran (green) flowing through a vascular network (red) in the presence of HCT116 tumor cells (blue). Lower panel shows 70 kDa FITC-dextran flowing through 3 VMTs connected in series.

SUPPLEMENTARY FIG. 4

Collagen synthesis, vascular development and tumor growth rate of two different CRC cell lines (SW620 and HCT116). The same number of SW620 and HCT116 tumor cells were co-cultured with EC and stromal cells. Indicated parameters were determined at day 7 of culture. Image J was used for tumor growth quantification. Total vessel length over

time was quantified using AngioTool. Vessel diameter was quantified using the MATLAB subroutine (RAVE), and Collagen I content was determined using second harmonic generation imaging as described in Material and Methods. Error bars show mean ± s.d of 3 replicates ($p < 0.05$).

SUPPLEMENTARY FIG. 5

(a) Dose response curves for 3 FDA-approved anti-cancer agents, 5-FU, Vincristine and Sorafenib, on HCT116 cells. VMTs or cells growing in 2D were exposed to the drugs for 48 h, and tumor cell number and viability were then assessed, either by fluorescence intensity measurement or XTT assay. Data are normalized to time zero of drug exposure and shown as percentage of control. Three replicates per experiment, error bars show mean ± s.d (n = 3- 7) ($p < 0.05$, 2D vs VMT)

(b) Calculated IC50 values for 4 FDA-approved anti-cancer drugs, including the drugs shown in (a) for both 2D and 3D cultures.

(c) Tumor drug screening comparison between two CRC that carry different mutations. HCT116 (CTNNB1, CDKN2A, KRAS, PIK3CA, MLH1) and SW620 (KRAS, MAP2K4, TP53, MYC, SMAD4) VMTs were first exposed to drugs between days 6 and 8 and cultured for an additional 96 h. Drugs were removed from the media at 48 h. Data are normalized to first day of drug exposure and are shown as percentage of control. Three replicates per experiment, error bars show mean ± s.d (n = 2- 4) (*$p < 0.05$ vs control; § $p < 0.05$ HCT116 vs SW620).

(d) 5-FU dose response comparison between HCT116 and SW620. Data are normalized to first day of drug exposure and are shown as percentage of control. Error bars show mean ± s.d from 3 replicates (n = 2) ($p < 0.05$).

SUPPLEMENTARY FIG. 6

Effect of the anti-vascular agents Linifanib (100 nM) and Pazopanib (100 nM) in the VMO. VMOs were exposed to the drug at day 5 and cultured for an additional 96 h. Drug was then removed, and the VMO were cultured again for an additional 96 h.

SUPPLEMENTARY FIG. 7

(a) Effect of Pazopanib on vessel diameter in the VMO. VMOs were exposed to drug from day 4 to 6 and then cultured for an additional 96 h. Data are normalized to day of first drug exposure and are shown as percentage of control. Quantification was performed as described in Materials and Methods. Three replicates per experiment, error bars show mean ± s.d (n = 3) ($p < 0.05$ vs control).
(b) Effect of FOLFOX on tumor growth and total vessel length. Three replicates per experiment. Error bars show mean ± s.d (n = 2) ($p < 0.05$ vs control). Data shown are from the same experiment presented in Figure 2c.

SUPPLEMENTARY FIG. 8

(a-e) Dose response curves of several anti-cancer agents that differentially target HCT116 tumor growth or vasculature in the VMT platform. VMTs were exposed to drug after 4 to 7 days in culture and then monitored for an additional 96 h. Oxaliplatin, Vincristine, and 5-FU were removed from the media at 48 h. Pazopanib and Sorafenib were present for the full 96 h. Data are normalized to time of first drug exposure and are shown as percentage of control. Three replicates per experiment. Error bars show mean ± s.d (n = 2- 5) ($p < 0.05$ tumor growth vs total vessel length).

SUPPLEMENTARY FIG. 9

(**a**) Representative FLIM imaging from a VMO. Lentivirally-transduced EC (red) overlaid onto the brightfield image (left) and alone (center left). Two-photon (2PE) microscopy showing NADH average fluorescence intensity (center) and FLIM NADH map (center right) from the same tissue chamber. NADH FLIM phasor distribution used to create the free/bound NADH color scale (right). (Scale bar 100 μm).

(**b-c**) Chemical disruption of metabolism in the VMO. (b) NADH FLIM map of the tissue chamber before and after 80min of KCN exposure. (c) Quantification of the response of cells to KCN showing poisoning of mitochondrial function by KCN. Shown are mean ± s.d of 3 replicates ($p < 0.05$).

SUPPLEMENTARY FIG. 10

Phasor scatter plot showing a significantly different ratio of free-bound NADH for the vasculature in the absence of flow compared to the presence of flow. Data were obtained 72h after the flow was reduced. Error bars show mean ± s.d ($p < 0.05$).

SUPPLEMENTARY FIG. 11

(a) MCF-7 tumor (red) within a VMT and corresponding 2PE-FLIM map showing the tumor cells and surrounding stroma. (b) NADH FLIM phasor distribution of the same tumor color-coded within the tumor only. Note heterogeneous metabolic profile of the tumor.

SUPPLEMENTARY VIDEOS

(**SV1**) 70 kDa FITC-dextran perfusion through 3D VMO.

(**SV2**) 3D reconstruction showing the interaction between microvessels and HCT116 tumor cells in the VMO. Lentivirally-transduced HCT116 (green) and EC (red) were visualized by confocal microscopy and the movie was created from 37 z-slices acquired every 5 µm (Scale bar 200 µm).

S1

a.

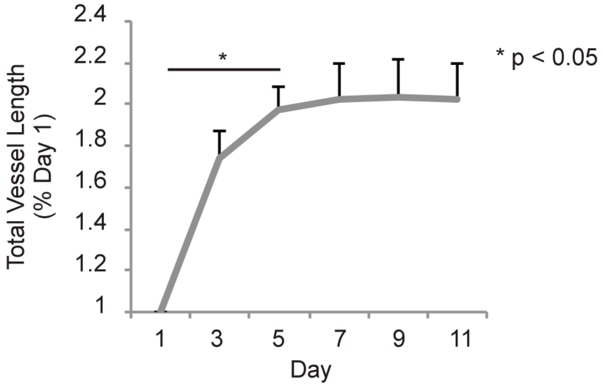
Total Vessel Length
(% Day 1)
2.4
2.2
2
1.8
1.6
1.4
1.2
1
1
3
5
7
9
11
Day
*
* p < 0.05

b.

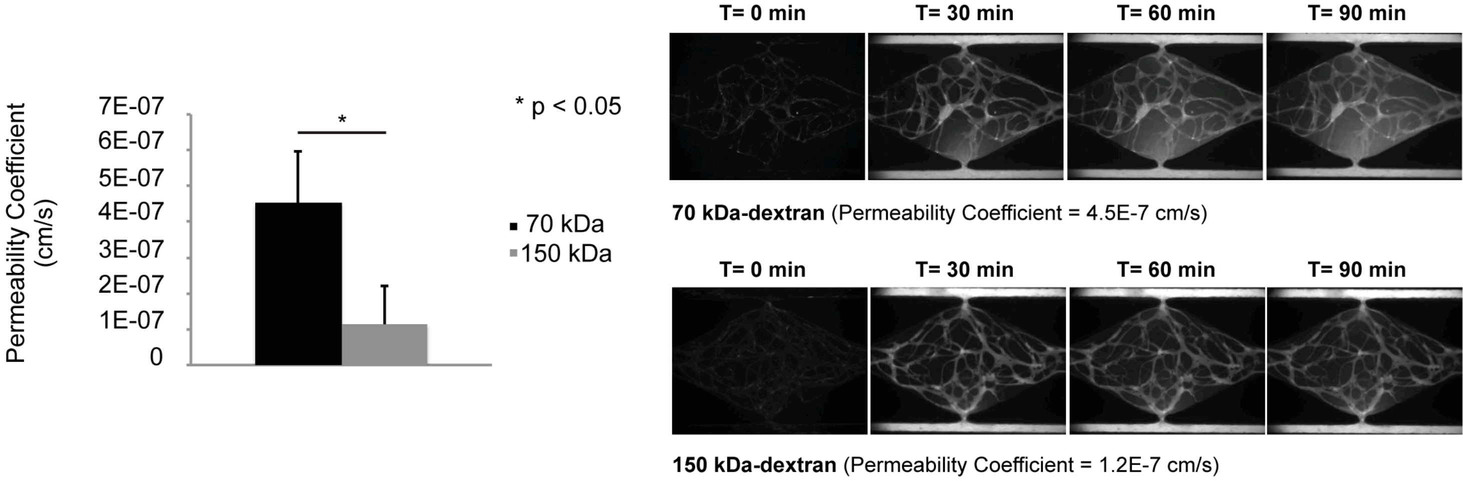
Permeability Coefficient
(cm/s)
7E-07
6E-07
5E-07
4E-07
3E-07
2E-07
1E-07
0
*
* p < 0.05
70 kDa
150 kDa
T= 0 min
T= 30 min
T= 60 min
T= 90 min
70 kDa-dextran (Permeability Coefficient = 4.5E-7 cm/s)
T= 0 min
T= 30 min
T= 60 min
T= 90 min
150 kDa-dextran (Permeability Coefficient = 1.2E-7 cm/s)

c.

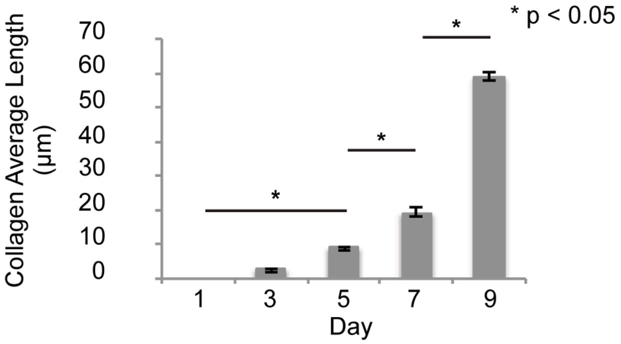
Collagen Average Length
(µm)
70
60
50
40
30
20
10
0
1
3
5
7
9
Day
*
*
*
* p < 0.05

S2

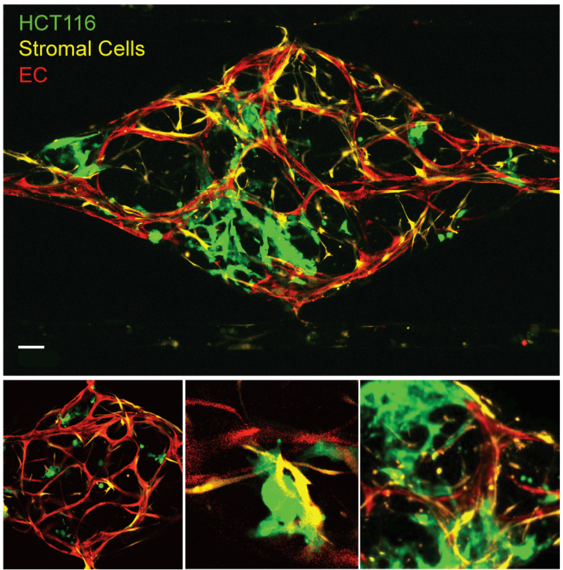
HCT116
Stromal Cells
EC

S3

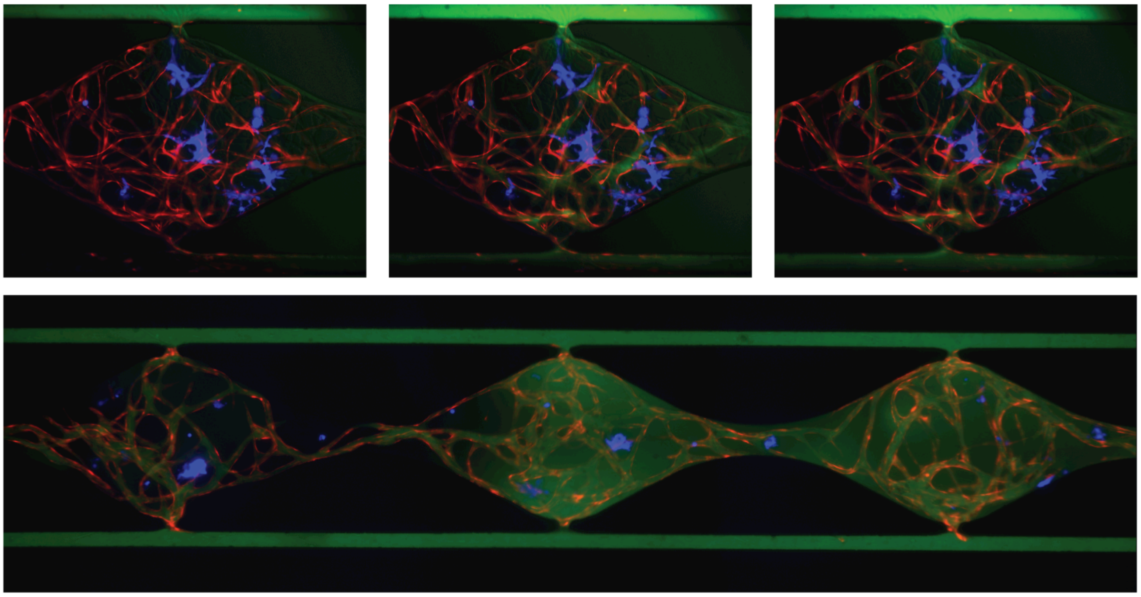

S4

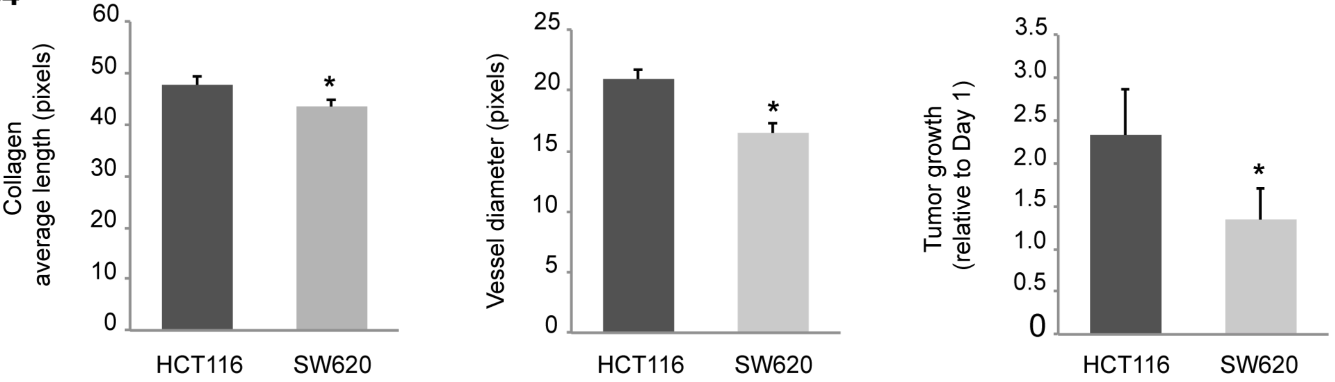

S5

a.

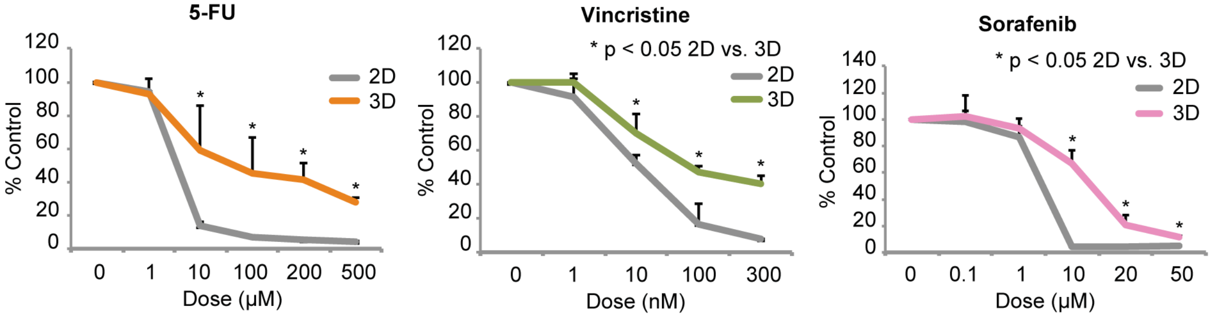

b.

Compound	2D	3D-VMT
5-FU	3.4 µM	56.1 µM
Oxaliplatin	0.7 µM	7.4 µM
Vincristine	9.3 nM	96 nM
Sorafenib	3.5 µM	8.0 µM

c.

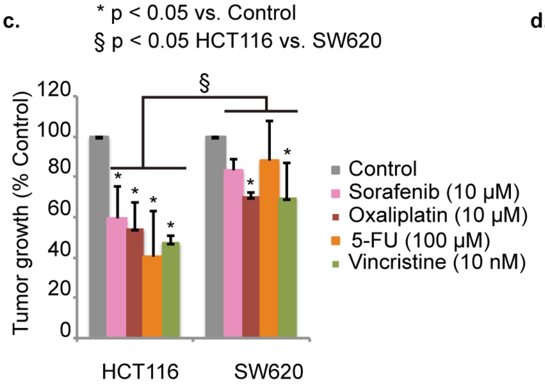

d.

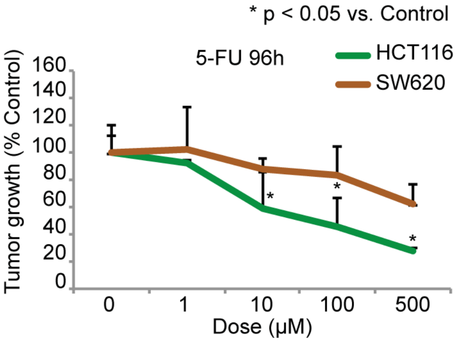

S6

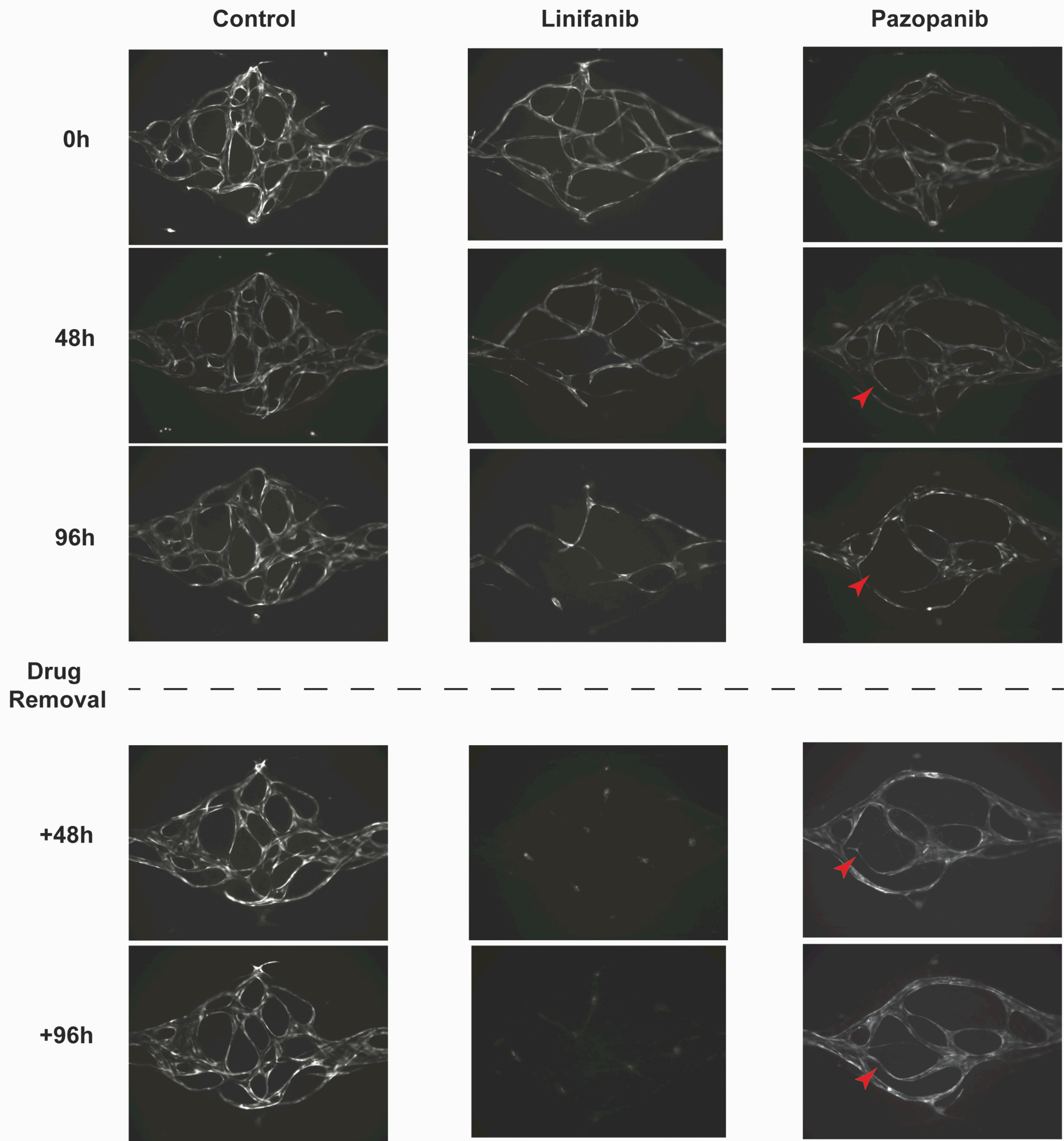
Control
Linifanib
Pazopanib
0h
48h
96h
Drug
Removal
+48h
+96h

S7

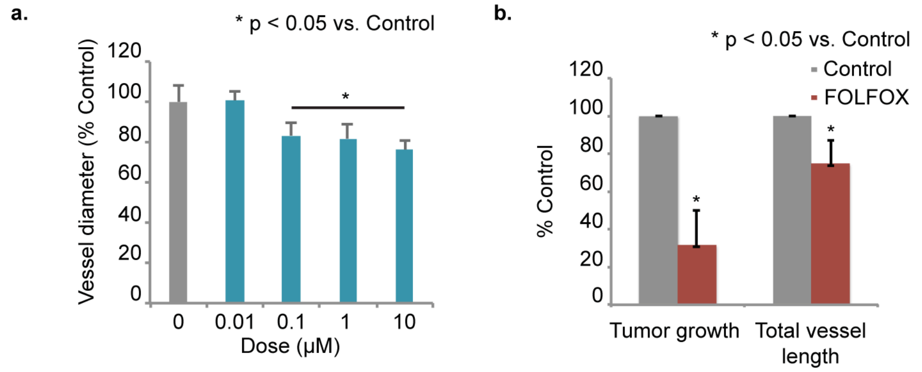
a.
* p < 0.05 vs. Control
Vessel diameter (% Control)
120
100
80
60
40
20
0
*
0
0.01
0.1
1
10
Dose (μM)
b.
* p < 0.05 vs. Control
Control
FOLFOX
% Control
120
100
80
60
40
20
0
*
*
Tumor growth
Total vessel length

S8

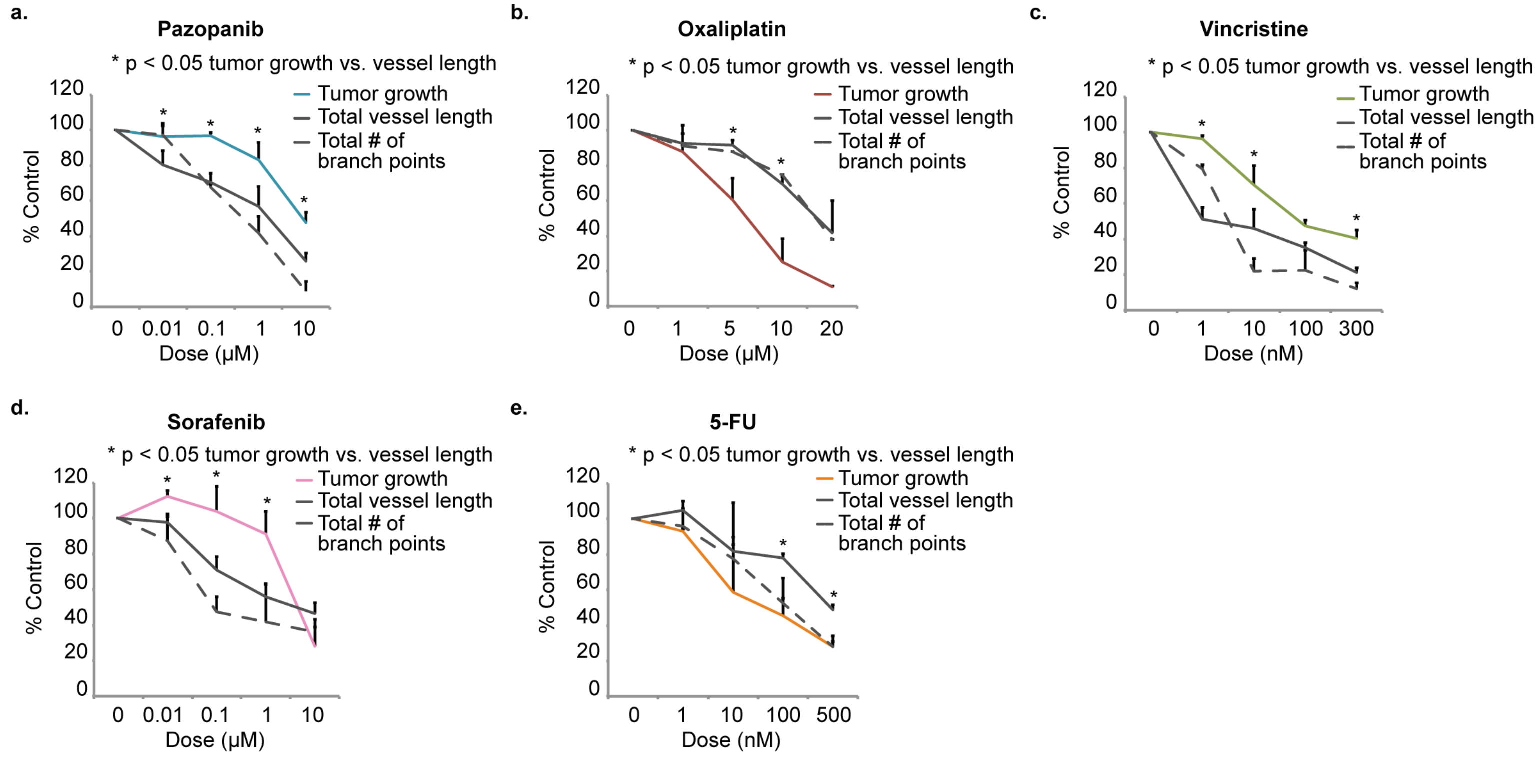
a.
Pazopanib
* p < 0.05 tumor growth vs. vessel length
Tumor growth
Total vessel length
Total # of branch points
% Control
120
100
80
60
40
20
0
0
0.01
0.1
1
10
Dose (μM)
b.
Oxaliplatin
* p < 0.05 tumor growth vs. vessel length
Tumor growth
Total vessel length
Total # of branch points
% Control
0
1
5
10
20
Dose (μM)
c.
Vincristine
* p < 0.05 tumor growth vs. vessel length
Tumor growth
Total vessel length
Total # of branch points
% Control
0
1
10
100
300
Dose (nM)
d.
Sorafenib
* p < 0.05 tumor growth vs. vessel length
Tumor growth
Total vessel length
Total # of branch points
% Control
0
0.01
0.1
1
10
Dose (μM)
e.
5-FU
* p < 0.05 tumor growth vs. vessel length
Tumor growth
Total vessel length
Total # of branch points
% Control
0
1
10
100
500
Dose (nM)

S9

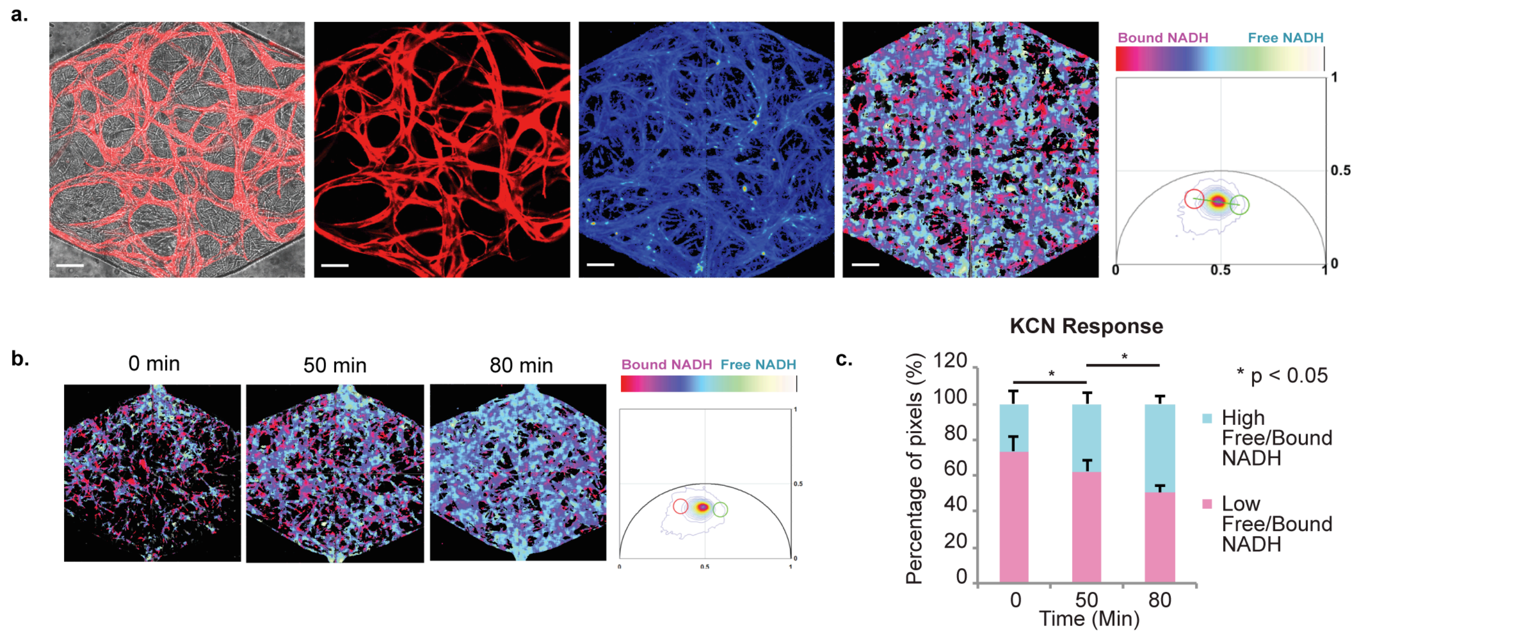
a.
Bound NADH
Free NADH
1
0.5
0
0
0.5
1
b.
0 min
50 min
80 min
Bound NADH
Free NADH
c.
KCN Response
* p < 0.05
Percentage of pixels (%)
120
100
80
60
40
20
0
*
*
High Free/Bound NADH
Low Free/Bound NADH
0
50
80
Time (Min)

S10

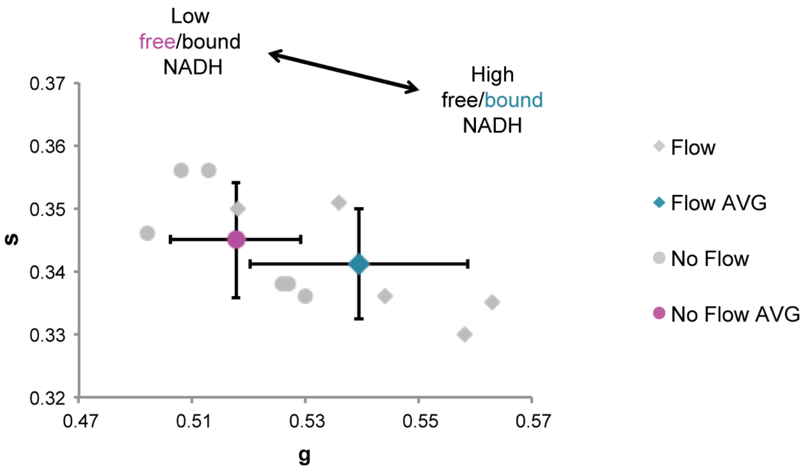
Low
free/bound
NADH
High
free/bound
NADH
0.37
0.36
0.35
0.34
0.33
0.32
s
0.47
0.51
0.53
0.55
0.57
g
Flow
Flow AVG
No Flow
No Flow AVG

S11

a.

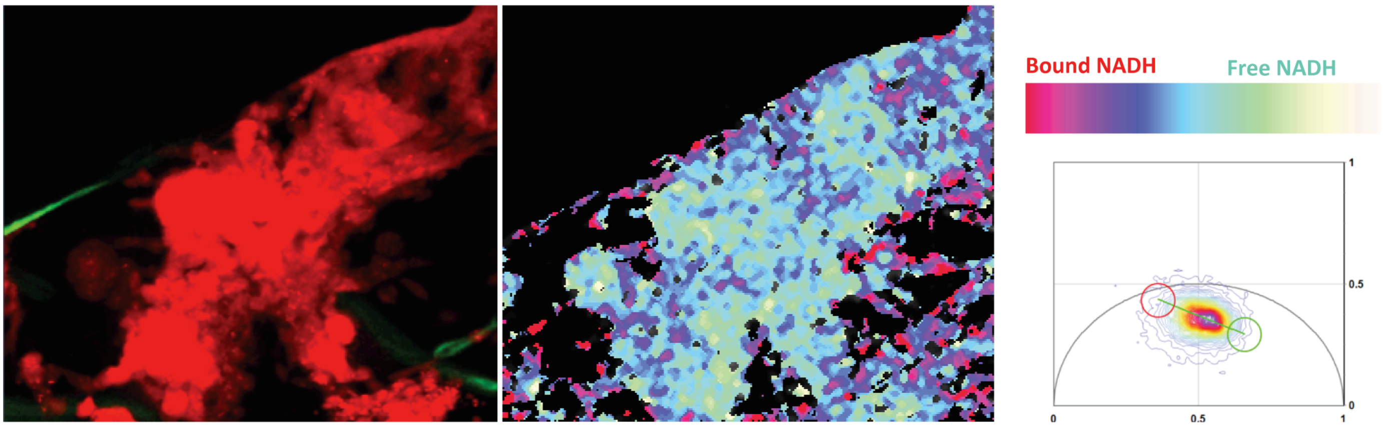
Bound NADH
Free NADH
1
0.5
0
0
0.5
1

b.

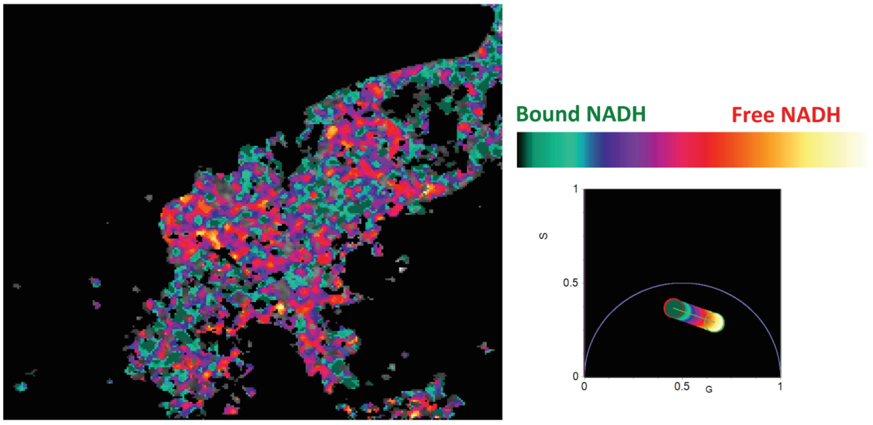
Bound NADH
Free NADH
1
S
0.5
0
0
0.5
G
1
